# Supplementary material for: Assessing the Factor Structure of the Farm/Ranch Stress Inventory in a Sample of LGBTQ+ Farmers Across the United States
Source: Int J Environ Res Public Health. 2025 Dec 23;23(1):22. doi: 10.3390/ijerph23010022 (PMC12840779; doi:10.3390/ijerph23010022)

Supplemental Table S1. Comparison between original FRSI items and factors with current analysis.

**Item**	**Original FRSI** [3]	**Current Analysis**
Not enough cash/capital for unexpected problems (illnesses, health care, breakdowns, other emergencies)	Finances	Family and finances
Financing for retirement	Finances	Family and finances
Not enough money for day to day expenses (purchases, repairs, parts, fence and building maintenance, etc.)	Finances	Family and finances
High debt load	Finances	Family and finances
Working with bankers and loan officers	Finances	Long-term farming viability
Health care costs (direct costs and/or cost of insurance)	Government and external stress	Family and finances
Taxes (high taxes, figuring taxes, etc.)	Government and external stress	Family and finances
Government farm price supports	Government and external stress	Long-term farming viability
Government export policy	Government and external stress	Long-term farming viability
Outsiders not understanding the nature of farming/ranching	Government and external stress	Cultural isolation
Limited social interaction opportunities	Isolation	Social and safety dynamics
Lack of close neighbors	Isolation	Social and safety dynamics
Distance from doctors or hospitals	Isolation	Logistical circumstances
Distance from shopping centers/schools/recreation, etc.	Isolation	Logistical circumstances
Market prices for your crops/livestock	Operations stressors	Family and finances
Problems with machinery (purchases, repairs, breakdowns)	Operations stressors	Operational challenges/ sustainability
Problems with livestock or crops (illness, disease, noxious weeds, rodents)	Operations stressors	Operational challenges/ sustainability
Operating hazardous machinery	Operations stressors	Long-term farming viability
Farm/ranch accidents and injuries	Operations stressors	Social and safety dynamics
The weather (inadequate/or too much rainfall, snow, hail, etc.)	Operations stressors	Logistical circumstances
Balancing the many roles I perform as a family member and a farmer/rancher	Work stressors	Family and finances
Not enough time to spend together as a family in recreation	Work stressors	Family and finances
Not having the people power to operate the farm/ranch	Work stressors	Operational challenges/ sustainability
Having too much work for one person	Work stressors	Operational challenges/ sustainability
Concern over the future of the farm/ranch	Work stressors	Operational challenges/ sustainability
Working with extended family members in the farm/ranch operation (parents, in-laws, children)	Work stressors	Social and safety dynamics
Seasonal variations in workload (planting season, harvest, calving time, marketing time, etc.)	Work stressors	Logistical circumstances
Dealing with non-relative help (incompetent help, finding good help, supervising help)	Work stressors	Cultural isolation

Supplemental Figure S1. Scree plot.

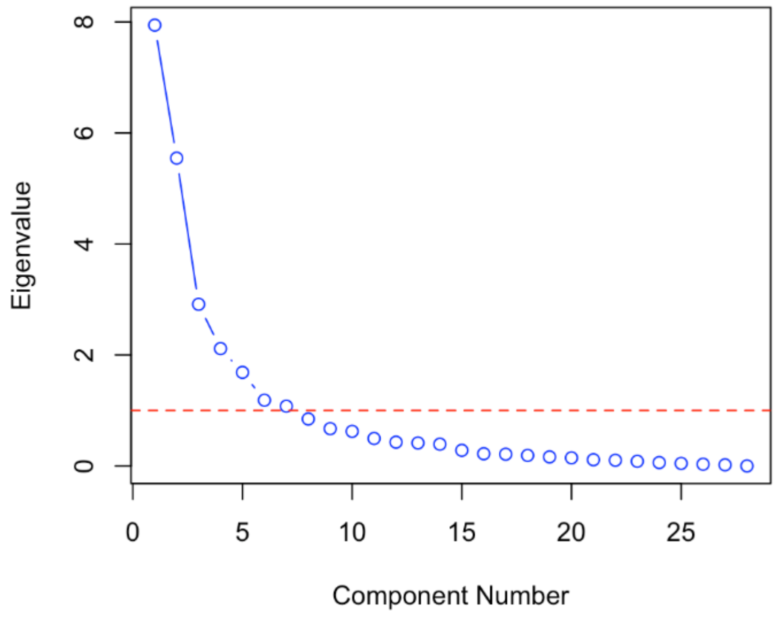

Supplement: Supplementary file 1 [file ijerph-23-00022-s001.zip › ijerph-3924233-supplementary.pdf]
